# Exploring naturally occurring clinical subgroups of post-traumatic headache

**DOI:** 10.1186/s10194-020-1080-2

**Published:** 2020-02-07

**Authors:** T. L. H. Chan, Y. W. Woldeamanuel

**Affiliations:** Division of Headache & Facial Pain, Department of Neurology & Neurological Sciences, Stanford University, Palo Alto, California USA

**Keywords:** Post-traumatic headache, Post-traumatic headache disorder, Acute post-traumatic headache, Persistent post-traumatic headache, Headache attributable to traumatic injury to the head and/or neck

## Abstract

**Objective:**

To explore naturally occurring clinical subgroups of post-traumatic headache.

**Background:**

Persistent post-traumatic headache (PTH) is defined as a headache developing within 7 days of an injury that lasts for greater than 3 months. However, there is no evidence available from the International Classification of Headache Disorders (ICHD) based classification between persistent and acute PTH based on clinical phenotypes.

**Methods:**

We conducted a retrospective study using the Stanford Research Repository Cohort Discovery Tool. We reviewed 500 electronic patient charts between January 2015 to September 2019 using inclusion criteria of adults older than 18 years with a diagnosis of PTH. The following variables were extracted from each patient’s chart: diagnosis of PTH as dependent variable, and predictor variables as age, sex, history of migraine, loss of consciousness during head injury, pre-existing psychological history, duration of PTH and new PTH-associated comorbidities (e.g. new onset vertigo, post-traumatic stress disorder). Logistic regression was employed to identify clinical phenotypes predicting persistent PTH. All predictor variables were tested in one block to determine their predictive capacity while controlling for other predictors in the model. Two-step cluster analysis was conducted to identify naturally occurring PTH subgroups.

**Results:**

A total of 300 patients were included (150 acute, 150 persistent PTH) with a median age of 47 years (IQR 31, 59) and female: male ratio of 2.7:1. Two hundred patients were excluded due to misdiagnoses. Pre-existing psychological history (standardized beta 0.16), history of migraine (0.20), new PTH-associated comorbidities (0.23) and medication overuse (0.37) statistically significantly predicted the presence of persistent PTH (*p* <  0.0001). Clustering analysis revealed PTH subgrouping comparable to ICHD-based classification: 140 patients in Cluster 1 (76% persistent PTH) and 160 patients in Cluster 2 (83% acute PTH). Four distinct clusters were found within persistent PTH.

**Conclusion:**

Pre-existing psychological history, history of migraine, new PTH-associated comorbidities and medication overuse predicted the occurrence of persistent PTH as well as two naturally occurring PTH clusters correlating to acute and persistent PTH. Management emphasis should focus on these phenotypes.

## Introduction

Headache attributed to traumatic injury to the head [1] also known as post-traumatic headache (PTH) is a common condition following injury to the head and/or neck. The prognosis is generally favorable with most cases resolving within 3–6 months of the inciting injury [2]. However, it is reported that 18–22% of PTH last for more than 1 year [3].

PTH is a poorly understood entity. According to the International Classification of Headache Disorders-3 (ICHD-3): It is defined as any headache related to a traumatic injury to the head and/or neck with headache being reported within 7 days [1]. Little is known about the pathophysiology of PTH: A number of factors have been suggested including microglial activation in the brain parenchyma, dural inflammation related to mast cell degranulation with sensitization of pain pathways, injury to the extracranial tissues and direct damage to neuronal and brain structures [4].

The diagnosis of acute versus persistent PTH is based on an arbitrary cutoff selection of 3 months of headache duration, greater than 3 months for persistent PTH and lesser than 3 months for acute PTH [1]. Limited evidence has looked into the factors associated with the transformation of acute to persistent PTH. A prior population-based study identified that history of traumatic brain injury, being injured under the influence of alcohol, and history of acute PTH were predictors for persistent PTH [5]. PTH is also associated with somatic, cognitive and psychological symptoms [6]. It is known that there is a bidirectional association between headache and psychiatric disorders [7, 8]. Anxiety, depression, affective temperamental dysregulation and suicidal behavior may be seen in patients with chronic headache disorders [6, 8, 9]. In the diagnosis of PTH, the possibility of co-occurring medication overuse headache (MOH) is an important consideration as well [10]. Based on this information, we wanted to test the hypothesis that exposure to clinical predictors, such as medication overuse and psychological symptoms are associated with persistent PTH compared to acute PTH. In addition, we hypothesized that there exists naturally occurring heterogeneous clusters within the persistent PTH group of patients.

In this hospital-based study, we explored the clinical predictors that may be more likely associated with persistent versus acute PTH. Identifying potential clinical predictors may have treatment implications and provide a plausible explanation as to why some patients develop persistent headaches after an injury to the head and/or neck. In addition, we conducted clustering analysis to identify naturally-occurring subgroups of PTH and compare them with ICHD-3 classification of acute versus persistent.

## Methods

### Study design

This was a case-referent retrospective chart review determining clinical predictors which accounted for persistent PTH (cases) in comparison to acute PTH (referents).

### Study setting

Charts were identified using the Stanford Research Repository Cohort Discovery (STARR) Tool - an online tool designed for chart identification and review. Search terms included “post-traumatic headache” and “headache attributed to traumatic injury to the head and/or neck” and “concussion” and “traumatic brain injury”. Patients were seen at Stanford-affiliated clinics i.e. Stanford Headache Clinic, Stanford Neurology Clinic, Stanford Pain Clinic, Stanford Concussion Clinic*.* The study period spanned between January 1, 2015 to September 31, 2019.

### Inclusion and exclusion criteria

A total of 500 electronic patient charts were reviewed. Inclusion criteria were adults aged 18 years and older with a diagnosis of PTH. Children younger than 18 years, patients without PTH diagnosis, and charts with limited information were excluded. Children were excluded since confounding effect from the developing brain will make it challenging to interpret results from our study design.

### Data extraction

In addition to PTH diagnosis (acute and persistent), the following variables were extracted from each patient’s chart: age, sex, history of migraine, loss of consciousness during head injury, cause of head injury (e.g. fall, being hit with an object, car accident), pre-existing psychological history, duration of PTH, new PTH-associated comorbidities (e.g. new onset vertigo, post-traumatic stress disorder), and medication overuse. Operational definition of medication overuse was employed based on ICHD-3 criteria [1] for frequency of medications overused in medication overuse headache.

### Sample size estimation

Sample size was estimated a priori using linear multiple regression on *F* tests. A total of 295 patients was required to achieve 80% power involving 8 predictor variables with α error probability of 0.05 and small effect size f^2^ of 0.05. The final sample size was made 300 patients. G*Power 3.1 (Universität Düsseldorf) software was used to compute sample size [11].

### Ethical approval

This study received approval from Stanford University Institutional Review Board (eProtocol#: 52404, IRB 61, Registration 4947).

### Statistical analysis

Descriptive and inferential statistics were used to describe and interpret data, respectively. Since our study was a retrospective design, odds ratio (OR) statistics were used to measure the odds of having persistent versus acute PTH in those presenting with different clinical variables (e.g. history of migraine, pre-existing psychological history, medication overuse, new PTH-associated comorbidities). Where OR was inflated, relative risk (RR) was used. Logistic regression was employed to identify clinical phenotypes predicting persistent PTH. Predictor variables (age, sex, loss of consciousness, history of migraine, pre-existing psychological history, new PTH-associated comorbidities, repeated head injuries, medication overuse) were tested in one block to determine their predictive capacity while controlling for other predictors in the model. The regression model’s goodness-of-fit was tested using Cox & Snell R [2], Nagelkerke R [2], and Hosmer and Lemeshow test. Significance threshold was corrected for multiple testing by dividing two-tailed *p*-value of 0.05 to 8, resulting in a new *p*-value of 0.006. In addition, two-step cluster analysis was utilized to identify naturally occurring PTH classification. Two-step cluster analysis was selected since the data were mostly categorical. Clustering criterion was Schwarz’s Bayesian Criterion, and the log-likelihood distance measure was applied. Number of clusters were determined automatically. Continuous variables (e.g. age) were normalized. Missing data were handled by listwise deletion. SPSS version 21 [12] was used for statistical analysis.

## Results

### Included and excluded patients

Of the 500 PTH patient charts screened, 300 were included in the final study sample involving 150 acute and 150 persistent PTH. Median age of the total included sample was 47 years (IQR 31, 59) and female:male ratio was 2.7:1. Of the 200 patients excluded, there was limited information on 95 (47.5%) patients, 16 (8%) had migraine only, and the remaining 89 (44.5%) had a different diagnosis (e.g. cerebrospinal fluid leak, brain tumor, sinus infection, intracranial abscess, meningitis, cerebral aneurysm).

### Comparison between acute and persistent PTH patients (Table 1)

There was no statistically significant difference in median age between the acute (44 years, IQR 28, 59) and persistent (50 years (36, 58)) PTH group (Mann-Whitney test, *p* = 0.16). Similarly, the female:male ratio was comparable between the acute (2.8) and persistent (2.5) PTH groups with no statistically significant difference (chi-squared test, *p* = 0.60). Median duration of PTH was 0.7 months (IQR 0.23, 1) in acute and 24 months (12, 48) in persistent PTH patients (Mann-Whitney test, *p* <  0.0001). Fifty-nine (40%) persistent PTH patients had a history of migraine compared to only 8 (5%) acute PTH patients (RR = 2.4, 95% CI 2, 3; *p* <  0.0001). With the patients who were diagnosed with persistent PTH and a history of migraine, it was documented that they had at least 2 fold increase in frequency and/or intensity of their headaches after their injury. History of repeated head injury was seen in 37 (25%) of the persistent PTH patients compared to only 8 (5%) of the acute PTH patients (OR = 5.8, 95% CI 2.6, 13; *p* <  0.0001). Twelve (8%) acute PTH patients suffered loss of consciousness compared to 32 (21%) persistent PTH patients (OR = 3.1, 95% CI: 1.5, 6.3; *p* = 0.0016). Only 1 (0.007%) acute PTH patient had medication overuse in contrast to 58 (39%) patients in the persistent PTH group (RR = 2.6, 95% CI: 2.2, 3.0; *p* < 0.0001). Pre-existing psychological history (e.g. depression, anxiety, bipolar, post-traumatic stress disorder) was found in 27 (18%) patients of the acute PTH group compared to 78 (52%) patients in the persistent PTH (OR = 5, 95% CI 3, 8.3; *p* < 0.0001). New PTH-associated comorbidities (e.g. vertigo, neck pain, imbalance) were seen in 103 (69%) acute PTH patients compared to 147 (98%) patients in the persistent PTH group (RR = 9.8, 95% CI 3.3, 29.5; *p* < 0.0001). All patients with acute PTH had complete resolution of their headache within 3 months of headache onset. Only 5 persistent PTH patients had complete resolution of their headache within a median duration of 9 months (IQR 6, 12). Overall, there was a 5% missing data which was excluded from analysis.

**Table 1:** Clinical features of acute and persistent PTH patients included in the study

Clinical Variables	Acute PTH (*n* = 150)	Persistent PTH (*n* = 150)	Statistical Difference
Age, years: median (IQR)	44 (28, 59)	50 (36, 58)	Mann-Whitney *p* = 0.16
Female:Male ratio	2.8	2.5	Chi-squared *p* = 0.60
Duration of PTH, months: median (IQR)	0.7 (0.23, 1)	24 (12, 48)	**Mann-Whitney** ***p*** **< 0.00001**
History of migraine: *n* (%)	8 (5%)	59 (40%)	**RR = 2.4** **95% CI 2, 3** ***p*** **< 0.0001**
Previous head injury: *n* (%)	8 (5%)	37 (25%)	**OR = 5.8** **95% CI 2.6, 13** ***p*** **< 0.0001**
Loss of consciousness: *n* (%)	12 (8%)	32 (21%)	**OR = 3.1** **95% CI: 1.5, 6.3** ***p*** **= 0.0016**
Medication overuse: *n* (%)	1 (0.007%)	58 (39%)	**RR = 2.6** **95% CI: 2.2, 3.0** ***p*** **< 0.0001**
Pre-existing psychological history: *n* (%)	27 (18%)	78 (52%)	**OR = 5** **95% CI 3, 8.3** ***p*** **< 0.0001**
New PTH-associated comorbidities: *n* (%)	103 (69%)	147 (98%)	**RR = 9.8** **95% CI 3.3, 29.5** ***p*** **< 0.0001**

*Abbreviations*: *IQR* interquartile range, *OR* odds ratio, *RR* relative risk, *CI* confidence interval. Statistically significant differences are displayed in bold

### Cause of head trauma (Table 2)

Motor vehicle accidents (MVA) was the cause of head trauma in 25 (16%) acute PTH patients compared to 46 (31%) patients in the persistent PTH group (OR = 2.2, 95% CI 1.3, 3.8; *p* = 0.0048). There was no statistically significant difference in fall injury as cause of head trauma between the acute and persistent PTH groups (OR = 0.8, 95% CI 0.5, 1.2; *p* = 0.35). Both acute and persistent PTH groups had similar prevalence of being hit by an object as cause of head trauma (OR = 0.8, 95% CI 0.5, 1.2; *p* = 0.26).

**Table 2:** Causes of head injury in the acute and persistent PTH group

Cause of Head Injury	Acute PTH	Persistent PTH	Statistical Difference
Motor Vehicle Accident: *n* (%)	25 (16%)	46 (31%)	**OR = 2.2** **95% CI 1.3, 3.8** ***p*** **= 0.0048**
Fall: *n* (%)	65 (44%)	57 (38%)	OR = 0.8 95% CI 0.5, 1.2 *p* = 0.35
Direct trauma to the head by an object: *n* (%)	60 (40%)	47 (31%)	OR = 0.8 95% CI 0.5, 1.2 *p* = 0.26

*OR* odds ratio, *CI* confidence interval. Statistically significant differences are displayed in bold

### Source of diagnosis (Table 3)

Diagnosis was made by headache specialists in the majority of persistent PTH patients (39%), while most of the acute PTH were diagnosed by emergency room physician (47%).

**Table 3:** Provider who diagnosed acute and persistent PTH

Provider	Acute PTH *n* (%)	Persistent PTH *n* (%)	Chi-squared *p-*value
Headache Specialist	1 (0.7%)	58 (39%)	**< 0.000001**
Neurology	4 (2.7%)	28 (19%)	**0.000007**
Pain Clinic	0 (0%)	18 (12%)	N/A
Family Medicine	29 (19.3%)	9 (6%)	**0.0005**
Internal Medicine	23 (15.3%)	5 (3.3%)	**0.0003**
Emergency Room Physician	70 (47%)	3 (2%)	**< 0.000001**
Sports Medicine, Physical Medicine and Rehabilitation, Concussion Clinic	23 (15.3%)	29 (19.3%)	0.36

Statistically significant differences are displayed in bold

### Logistic regression (Table 4)

Logistic regression showed that pre-existing psychological history (standardized beta 0.16), history of migraine (0.20), new PTH-associated comorbidities (0.23) and medication overuse (0.37) statistically significantly predicted the presence of persistent PTH (*p* < 0.0001). Duration of persistent PTH did not impact other clinical variables. Goodness-of-fit statistics showed the predictive capacity of the model was fit and appropriate (Cox & Snell R square 0.46, Nagelkerke R square 0.61, Hosmer and Lemeshow test *p* = 0.34).

**Table 4:** Logistic regression results

	β unstandardized	β standardized	OR (95% CI) standardized	*p*-value
Age	0.23	0.06	1.06 (0.98, 2.65)	0.175
Sex	−0.25	−0.06	0.94 (0.87, 2.39)	0.117
MigHis	**0.81**	**0.20**	**1.22 (1.11, 3.04)**	**0**
LOC	0.33	0.08	1.08 (1.00, 2.71)	0.056
PrevHI	0.65	0.16	1.17 (1.03, 2.80)	0.018
MO	**1.50**	**0.37**	**1.44 (1.18, 3.26)**	**0**
PPH	**0.65**	**0.16**	**1.17 (1.08, 2.94)**	**0**
NewCom	**0.96**	**0.23**	**1.26 (1.12, 3.06)**	**0**

Dependent variable was acute or persistent PTH, while independent variables were age, sex, history of migraine (MigHis), loss of consciousness during head injury (LOC), previous head injury (PrevHI), medication overuse (MO), pre-existing psychological history (PPH), and new PTH-associated comorbidities (NewCom). Variables in bold font were found to statistically significantly contribute to the prevalence of persistent PTH. Results are displayed using unstandardized and standardized regression coefficients (β), odds ratio (OR), and *p*-values

### Head imaging

In the persistent PTH patients, head imaging was done in 143 (95%) - of which 33 (23%) had findings (skull and/or facial bone fractures, subdural hematoma, subarachnoid and/or intracerebral hemorrhage, encephalomalacia), and the rest 110 (77%) had normal head imaging results. In the acute PTH patients, 75 (50%) had head imaging - of which 60 (80%) showed normal findings and the remaining 15 (20%) had skull and/or facial fractures, subdural hematoma, subarachnoid and/or intracerebral hemorrhage (Chi-squared = 0.27, *p* = 0.60).

### Two-step cluster analysis (Figs. 1 and 2)

Two-step cluster analysis results revealed 2 clusters of PTH i.e. 140 (46.7%) patients assembled in Cluster 1 and 160 (53.7%) patients in Cluster 2. 106 (76%) of Cluster 1 patients had persistent PTH while 133 (83%) of Cluster 2 patients had acute PTH. Cluster 1 PTH patients had higher prevalence of a history of migraine, medication overuse, pre-existing psychological history, new PTH-associated comorbidities, history of repeated head injury compared to Cluster 2 PTH patients. History of migraine, higher level of medication overuse and pre-existing psychological history ranked as the top 3 predictors of Cluster 1 from Cluster 2.

Four clusters of persistent PTH patients were identified. Cluster 1 had the lowest prevalence of a history of migraine. Cluster 2 featured the highest level of pre-existing psychological history, medication overuse, history of migraine, loss of consciousness, and the longest PTH duration (median 30 months). Cluster 3 had the shortest PTH duration (median 24 months) and the lowest prevalence of loss of consciousness. Cluster 4 exhibited the lowest prevalence of new PTH-associated comorbidities and head injuries. Pre-existing psychological history, medication overuse, and history of migraine ranked as the top 3 predictors of the clusters.

**Fig. 1:**
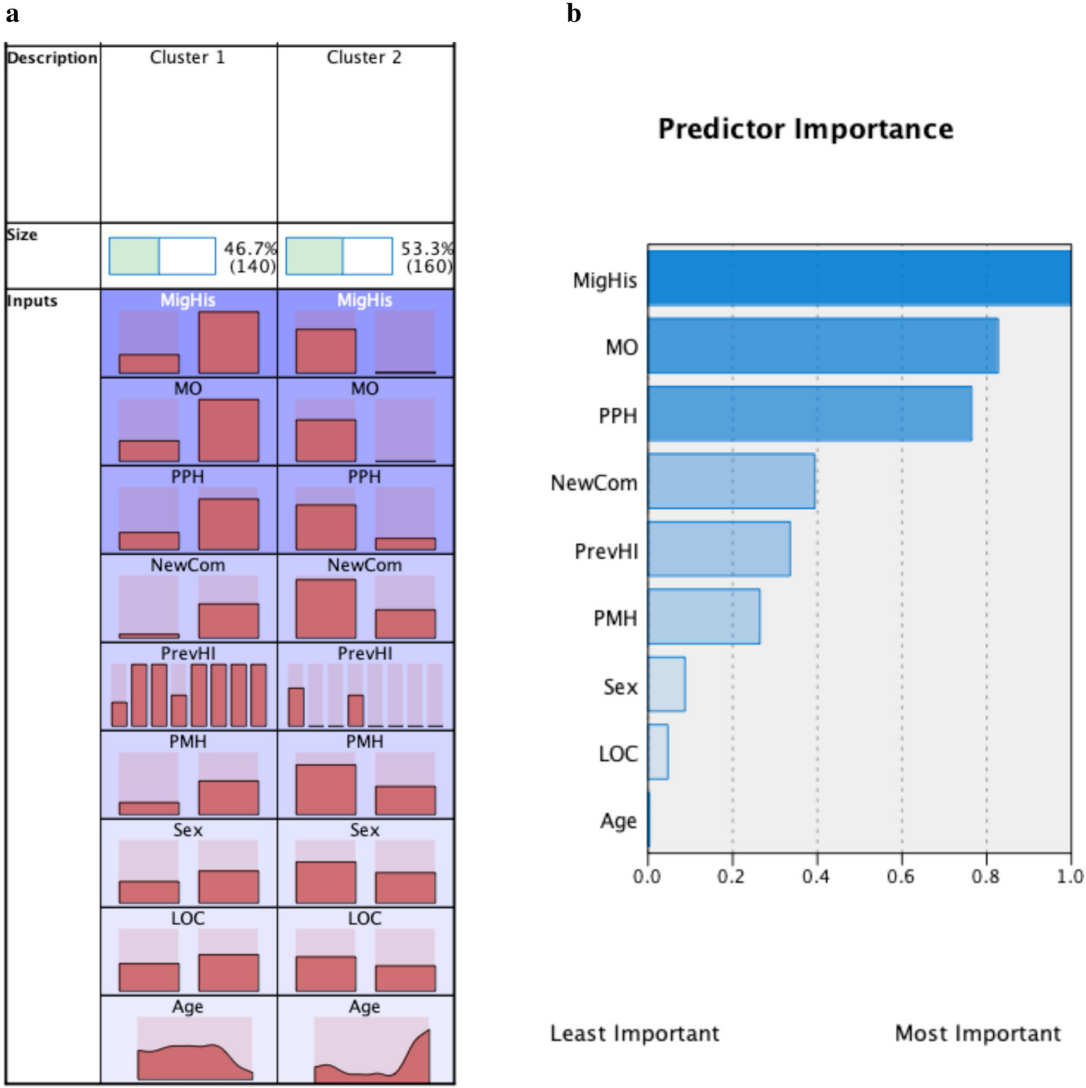
PTH clusters. Two PTH clusters were identified (**a**). Cluster 1 contained 140 (46.7%) patients and 160 (53.7%) patients assembled in Cluster 2 (**a**). One hundred and six (76%) Cluster 1 patients had persistent PTH while 133 (83%) of Cluster 2 patients had acute PTH. Compared to Cluster 2 patients, Cluster 1 PTH patients had higher prevalence of a history of migraine (MigHis), medication overuse (MO), pre-existing psychological history (PPH), new PTH-associated comorbidities (NewCom), history of repeated head injuries (PrevHI) compared to Cluster 2 PTH patients (**a**). The top 3 important predictors were a history of migraine, higher level of medication overuse and pre-existing psychological history (**b**). The bars in **a** show relative distribution of the different clinical variables - with the right bar in each cluster representing present clinical variable while the left bar is for absent variable. For example, the first row for history of migraine (MigHis) shows that Cluster 1 had relatively higher prevalence compared to Cluster 2. The seven bars in previous head injury (PrevHI) represent frequency of head injuries ranging from 0 (left) to 7 (right) under each cluster; there were more patients with more frequent head injuries in Cluster 1 compared to Cluster 2. Age distribution is shown ranging from 18 (left) to 90 (right) years. For sex, left bars represent males while right bars represent females

**Fig. 2:**
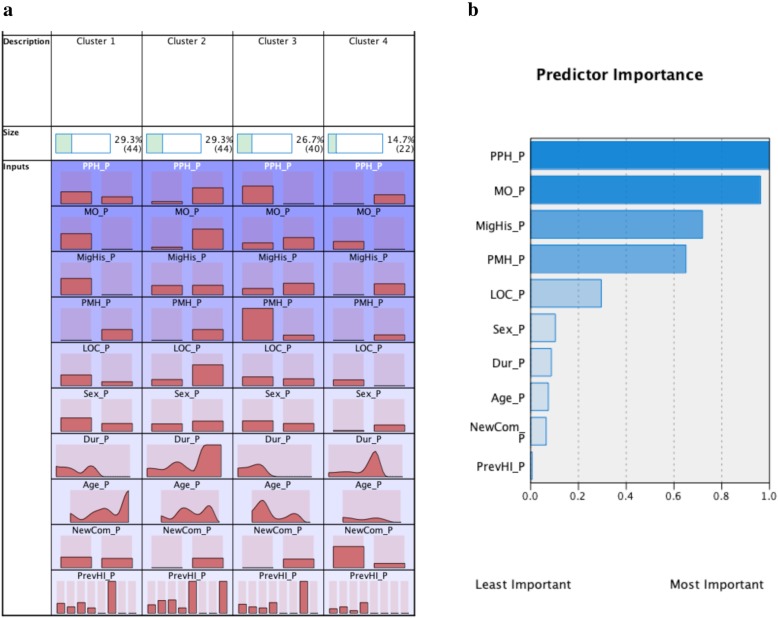
Clusters within persistent PTH patients. Four clusters of persistent PTH patients were identified **(a)**. Under each cluster column, the following clinical variables were coded as “present” and “absent”: PPH_P (previous psychological history in persistent PTH), MO (medication overuse), MigHis (migraine history), PMH (previous medical history), LOC (loss of consciousness), NewCom (new comorbidities). “Present” is depicted by the left bars under each cluster column, while “absent” is represented by the right bars. For sex, left bars represent males while right bars represent females. The seven bars in previous head injury (PrevHI) represent frequency of head injuries found ranging from 0 (left) to 7 (right). Age distribution is shown ranging from 18 (left) to 90 (right) years. Cluster 1 had the lowest prevalence of a history of migraine (i.e. highest bar for “absent” MigHis). Cluster 2 featured the highest level of pre-existing psychological history, medication overuse, history of migraine, loss of consciousness, and the longest PTH duration (median 30 months). Cluster 3 had the shortest PTH duration (median 24 months) and the lowest prevalence of loss of consciousness. Cluster 4 exhibited the lowest prevalence of new PTH-associated comorbidities and head injuries **(a)**. The top 3 important predictors in descending order were pre-existing psychological history, medication overuse, and history of migraine **(b)**

## Discussion

In this study, pre-existing psychological history, history of migraine, new PTH-associated comorbidities and medication overuse predicted the occurrence of persistent PTH. Previous studies have suggested that a previous history of headache, less severe injury, female gender and the presence of comorbid psychiatric disorders are associated with PTH [5, 7, 13–16].

However, these studies did not specify who made the diagnosis and the details on their previous headache history, including their headache diagnosis, duration, frequency and/or intensity. In our study, the diagnostic accuracy for the persistent PTH group was high since 58% of them were made by neurologists and headache specialists (Table 2). Our study reflects the real word setting where acute PTH is often seen and managed by primary care physicians including family medicine, internal medicine and emergency room physicians. When PTH becomes persistent and refractory to treatment, they are referred to specialty clinics. Based on the ICHD diagnostic criteria, PTH can only be diagnosed in the setting of migraine if patients have at least 2 fold increase in frequency and/or intensity of their headaches after their injury [1]. This information was documented within the persistent PTH group. Our study found that the duration of persistent PTH did not impact other clinical variables. This may be related to trigeminal neuroplasticity and central sensitization seen in prolonged PTH [17–19].

Our clustering analysis revealed two naturally occurring PTH clusters which highly correlated with ICHD-based acute versus persistent PTH classification. Cluster 1 represented the majority of persistent PTH while Cluster 2 represented the majority of acute PTH patients. A history of migraine, medication overuse, pre-existing psychological history, new PTH-associated comorbidities ranked as the top 4 classifying clinical variables in descending order. Cluster 1 phenotype was burdened with high levels of these 4 clinical variables compared to Cluster 2. That our clustering results mostly corroborated with ICHD-based classification provides evidence base for acute versus persistent PTH subgroups.

Our findings of four naturally occurring clusters within the persistent PTH group shows the presence of distinct persistent PTH profiles. This proves that not all persistent PTH patients are similar, and thus cannot be placed under an umbrella classification of “persistent PTH”. Some persistent PTH patients may have resolution within 2 years (i.e., Cluster 3) while others may have longer duration, higher psychological burden, and medication overuse (i.e., Cluster 2). Identifying these naturally occurring PTH clusters is important for providing personalized clinical management as well as for conducting precise clinical trials, since different patients may respond differently as per their baseline distinct cluster characteristics. There are a few possible pathophysiological mechanisms to explain the development of the four different clusters of persistent PTH: Cluster 1, in contrast to Cluster 2 was found to have the lowest migraine prevalence with low levels of psychological comorbidity and medication overuse while featuring moderate levels of new PTH-associated comorbidities may indicate uncontaminated phenotype of persistent PTH. Studying this phenotype may reveal distinct PTH-specific neuroanatomic regions involved e.g. dysfunctional inhibitory pathways which follow pericranial tissue injury [20]. Cluster 1 persistent PTH phenotype may explain whether PTH has a different mechanism compared to perturbed sensory processing and subcortical aminergic modulatory pathways described in migraine [21, 22]. Cluster 2 PTH patients may be exhibiting pronounced neuroinflammation, and increased peripheral and central sensitization, as shown by protracted migraine history and psychological comorbidities. Studies have shown that patients recovering from unconscious states can have long term chronic pain experiences due to aberrant limbic and trigemino-amygdalar pathways [23–27]. This may explain our finding of Cluster 3 patients featuring the shortest PTH duration along with the lowest prevalence of loss of consciousness. Likewise, Cluster 2 patients having correlation of higher prevalence of loss of consciousness and longer PTH duration supports this speculation. Cluster 4 patients seemed to signify the direct relationship between frequency of head injuries and new PTH-associated comorbidities. Cluster 4 persistent PTH patients may also be having increased natural tolerance to pain behavior as evidenced by the cluster’s relatively lower prevalence of psychological comorbidity, medication overuse, and migraine history.

The overuse of abortive headache medications may contribute to the chronicity of headache after a head injury. A study conducted at the Danish Headache Center showed that 42% of patients who fulfilled the criteria for PTH at the time of referral also fulfilled the criteria for MOH [10]. This may suggest a percentage of patients with persistent PTH may have MOH rather than true persistent PTH and refractory headache in persistent PTH may be partly caused by MOH. Prospective studies are required to explore if medication overuse is a confounding factor and/or play a role in promoting chronicity in patients with PTH. A previous published study demonstrated that migraine patients are more prone to develop MOH compared to patients with other headache types such as cluster headache [28]. This increased susceptibility may explain why migraine and medication overuse predicted the occurrence of persistent PTH in our study. Furthermore, continuous intake of acute headache medications may alter descending inhibitory pathways that are thought to be important mechanisms in PTH [20].

A previous published study showed that individuals with a history of headache, such as migraine were significantly more likely to report headaches both acutely and chronically after a traumatic head injury compared to those without a history of headaches [29]. This suggests that a history of headache may predispose patients in developing persistent PTH after a head injury. Although the pathophysiology of PTH is not completely understood, the proposed mechanism of impaired descending neuromodulation, activation of trigeminal and cervical afferents, neurometabolic changes, cortical spreading depression, calcitonin gene-related peptide dependent mechanisms and neuroinflammation overlap with the migraine entity [30]. One would suspect after further injury to the head and/or neck, the underlying process would be intensified.

Previous report has suggested that the occurrence of PTH after mild brain injury was not related to the type of injury [14]. However, in our study, motor vehicle accidents are associated with the persistent PTH group (Table 1). This may suggest the development of other comorbidities from a motor vehicle accident, such as post-traumatic stress disorder, vestibular dysfunction and neck injury. Patients within the two PTH groups had mild traumatic brain injury. Headache prevalence and severity have been reported to be greater in those with mild head injury compared to those with more severe head injury 13 . it is unclear why this inverse dose response relationship is seen, and further investigation is warranted.

Rarely does headache occur in isolation in closed head injury and other comorbidities are often seen. From our study, patients in the persistent PTH group suffered from multiple new PTH-associated comorbidities, including neck pain, vertigo, back pain, autonomic disturbance, anxiety, depression and cognitive impairment. Neck pain is one of the most common associated symptoms/comorbidities in the persistent PTH group: 70 out of the 150 patients (46%) had neck pain. The injured cervical structures could, in addition to causing neck pain, refer pain to the head due to a close relationship between the upper cervical inputs and the trigeminal system [31]. Hooten et al. has demonstrated significant reduction in headache frequency, intensity and neck pain after 12 months of exercise therapy for patients with a history of cervicogenic headaches, which may be part of the phenotype seen in PTH [32]. Thus, treatment on the neck, such as neck physical therapy may benefit PTH.

A pre-existing psychological history, including depression, anxiety, bipolar disease, post-traumatic stress orders were associated with the development of persistent PTH. Epidemiological and functional imaging studies suggest that a directional relationship exists between chronic pain and mental health disorders [32]. Stilling et al. has shown a significant reduction in depression rating and headache frequency after 1 month of repetitive transcranial magnetic stimulation (rTMS), and rTMS is an FDA approved treatment for depression [33]. The alteration of neurotransmitters such as serotonin and dopamine have a major role in pain modulation [32]. With better mental health, one would be able to be more active and practice good lifestyle routines. These measures can lead to a good clinical outcome in patients with headache [34, 35].

To note, two hundred patients were excluded in this study suggesting the diagnostic criteria of PTH may be an unfamiliar entity amongst healthcare providers. In addition, this may indicate STARR tool may give rise to false positive PTH identifications. The STARR identification was 60% accurate (300 out of 500 patients), which is an acceptable percentage based on a simple word-based patient search. Further education is required for providers who manage PTH, because a misdiagnosis may alter the management plan and have litigation implications.

Strengths of this study include application of robust statistical tests, with adequate goodness-of-fit regression models, adjustment for confounders, examination of multiple headache-related variables. More than 58% of the persistent PTH patients were diagnosed by neurologists or headache specialists (Table 2).

Our limitations included the following: The precise time of headache resolution or improvement was unavailable within the acute PTH group. Acute PTH results were based on patients not reporting headaches at their next physician visit (less than 3 months). Some information was not available including lifestyle routine changes, and details on allied health involvement and this was inherent to the retrospective study. Acute PTH was diagnosed mainly by general practitioners or emergency room physicians who may not be familiar with the diagnostic criteria of PTH. Assessment of pre-injury headache suffering may be influenced by recall problems. Our hospital-based study may not be representative of the general PTH population. Our results may not be valid for all age groups since we only studied participants from age greater than 18 years old. A causation could not be established from our results, rather association only. Although a previous clinical-based study in PTH patients reported higher prevalence of tension-type headache than migraine [10], tension-type headache was rarely documented or labeled as a pre-existing diagnosis in our study. Our speculation is that tension-type headache may not be a common referral to our center. Besides, tension-type headache is thought to be more common in community-based studies than in clinical-based studies [36, 37]. Hence, our clinical-based study setting may not give the true population burden of pre-existing tension-type headache in PTH patients.

## Conclusion and future direction

A pre-existing psychological history, history of migraine, new PTH-associated comorbidities and medication overuse predicted the occurrence of persistent PTH. Our study has raised a few interesting questions: Would there be a difference in clinical outcome between patients who have medication overuse versus no medication overuse in the development of PTH. Pre-existing psychological history, history of migraine, new PTH-associated comorbidities and medication overuse were found to be associated with persistent PTH. These clinical variables should be targeted as part of early treatment plan since managing these variables may impact PTH prognosis and recovery. Future prospective studies are needed to further validate our results. In addition, our study showed that data-driven classification can perform accurately correlating to ICHD-based PTH subclasses - hence providing evidence based for ICHD classification. In the future, machine learning tools can be developed based on our clustering results using clinical variables such as pre-existing psychological history, history of migraine, new PTH-associated comorbidities and medication overuse. Also, it could be useful to develop a specific score for predicting patients at risk of developing persistent PTH, instead of using non-specific assessment tools such as the Sports Concussion Assessment Tool (SCAT3) [38].

## Data Availability

The datasets analyzed during the current study are available from the corresponding author on reasonable request.

## References

[1] Headache Classification Committee of the International Headache Society (IHS) (2018). The International Classification of Headache Disorders, 3rd edition. Cephalalgia.

[2] Lane JC, Arciniegas DB (2002). Post-traumatic headache. Curr Treat Options Neurol.

[3] Theeler B, Lucas S, Riechers RG (2013). Post-traumatic headaches in civilians and military personnel: a comparative, clinical review. Headache.

[4] Mares C, Dagher JH, Harissi-Dagher M (2019). Narrative review of the pathophysiology of headaches and photosensitivity in mild traumatic brain injury and concussion. Can J Neurol Sci.

[5] Nordhaug Lena H., Linde Mattias, Follestad Turid, Skandsen Øystein Njølstad, Bjarkø Vera Vik, Skandsen Toril, Vik Anne (2019). Change in Headache Suffering and Predictors of Headache after Mild Traumatic Brain Injury: A Population-Based, Controlled, Longitudinal Study with Twelve-Month Follow-Up. Journal of Neurotrauma.

[6] Lucas S, Kobeissy FH (2015). Characterization and Management of Headache after mild traumatic brain injury. Brain Neurotrauma: molecular, neuropsychological, and rehabilitation aspects.

[7] Torelli P, Lambru G, Manzoni GC (2006). Psychiatric comorbidity and headache: clinical and therapeutical aspects. Neurol Sci.

[8] Pompili M, Serafini G, Di Cosimo D (2010). Psychiatric comorbidity and suicide risk in patients with chronic migraine. Neuropsychiatr Dis Treat.

[9] Serafini G, Pompili M, Innamorati M (2012). Gene variants with suicidal risk in a sample of subjects with chronic migraine and affective temperamental dysregulation. Eur Rev Med Pharmacol Sci.

[10] Baandrup L, Jensen R (2005). Chronic post-traumatic headache--a clinical analysis in relation to the international headache classification 2nd edition. Cephalalgia.

[11] Faul F, Erdfelder E, Buchner A (2009). Statistical power analyses using G*power 3.1: tests for correlation and regression analyses. Behav Res Methods.

[12] IBM Corp Released (2012) IBM SPSS statistics for windows, version 21.0. IBM Corp, Armonk

[13] Nordhaug LH, Hagen K, Vik A (2018). Headache following head injury: a population-based longitudinal cohort study (HUNT). J Headache Pain.

[14] Jouzdani SR, Ebrahimi A, Rezaee M (2014). Characteristics of posttraumatic headache following mild traumatic brain injury in military personnel in Iran. Environ Health Prev Med.

[15] Lieba-Samal D, Platzer P, Seidel S (2011). Characteristics of acute posttraumatic headache following mild head injury. Cephalalgia.

[16] Lange RT, Iverson GL, Rose A (2011). Depression strongly influences postconcussion symptom reporting following mild traumatic brain injury. J Head Trauma Rehabil.

[17] Navratilova Edita, Rau Jill, Oyarzo Janice, Tien Jason, Mackenzie Kimberly, Stratton Jennifer, Remeniuk Bethany, Schwedt Todd, Anderson Trent, Dodick David, Porreca Frank (2019). CGRP-dependent and independent mechanisms of acute and persistent post-traumatic headache following mild traumatic brain injury in mice. Cephalalgia.

[18] Mustafa G, Hou J, Tsuda S (2016). Trigeminal neuroplasticity underlies allodynia in a preclinical model of mild closed head traumatic brain injury (cTBI). Neuropharmacology.

[19] Defrin R (2014). Chronic post-traumatic headache: clinical findings and possible mechanisms. J Man Manip Ther.

[20] Defrin R, Schreiber S, Feingold Y (2011). Reduced pain modulation in patients with chronic post traumatic headache. Eur J Pain Suppl.

[21] Goadsby PJ, Holland PR, Martins-Oliveira M (2017). Pathophysiology of migraine: a disorder of sensory processing. Physiol Rev.

[22] Goadsby PJ (2012). Pathophysiology of migraine. Ann Indian Acad Neurol.

[23] Bernard JF, Besson JM (1990). The spino(trigemino) pontoamygdaloid pathway: electrophysiological evidence for an involvement in pain processes. J Neurophysiol.

[24] Morris JS, Ohman A, Dolan RJ (1998). Conscious and unconscious emotional learning in the human amygdala. Nature.

[25] Garcia-Larrea L, Bastuji H (2018). Pain and consciousness. Prog Neuro-Psychopharmacol Biol Psychiatry.

[26] Lukaszewicz A-C, Dereu D, Gayat E (2015). The relevance of pupillometry for evaluation of analgesia before noxious procedures in the intensive care unit. Anesth Analg.

[27] Bienvenu OJ, Gellar J, Althouse BM (2013). Post-traumatic stress disorder symptoms after acute lung injury: a 2-year prospective longitudinal study. Psychol Med.

[28] Paemeleire K, Bahra A, Evers S (2006). Medication-overuse headache in patients with cluster headache. Neurology.

[29] Hoffman JM, Lucas S, Dikmen S (2011). Natural history of headache after traumatic brain injury. J Neurotrauma.

[30] Ashina H, Porreca F, Anderson T (2019). Post-traumatic headache: epidemiology and pathophysiological insights. Nat Rev Neurol.

[31] Bartsch T, Goadsby PJ (2003). Increased responses in trigeminocervical nociceptive neurons to cervical input after stimulation of the dura mater. Brain.

[32] Hooten WM (2016). Chronic pain and mental health disorders: shared neural mechanisms, epidemiology, and treatment. Mayo Clin Proc.

[33] Stilling Joan, Paxman Eric, Mercier Leah, Gan Liu Shi, Wang Meng, Amoozegar Farnaz, Dukelow Sean P., Monchi Oury, Debert Chantel (2020). Treatment of Persistent Post-Traumatic Headache and Post-Concussion Symptoms Using Repetitive Transcranial Magnetic Stimulation: A Pilot, Double-Blind, Randomized Controlled Trial. Journal of Neurotrauma.

[34] Smitherman TA, Maizels M, Penzien DB (2008). Headache chronification: screening and behavioral management of comorbid depressive and anxiety disorders. Headache.

[35] Baskin SM, Lipchik GL, Smitherman TA (2006). Mood and anxiety disorders in chronic headache. Headache.

[36] GBD 2016 Headache Collaborators (2018). Global, regional, and national burden of migraine and tension-type headache, 1990–2016: a systematic analysis for the Global Burden of Disease Study 2016. Lancet Neurol.

[37] Chowdhury D (2012). Tension type headache. Ann Indian Acad Neurol.

[38] McCrory P, Meeuwisse WH, Aubry M, Cantu RC, Dvorak J, Echemendia RJ, Engebretsen L, Johnston KM, Kutcher JS, Raftery M, Sills A (2013). Consensu statement on concussion in sport—the 4th international conference on concussion in sport held in Zurich, November 2012. PM&R.

